# Prevalence and Virulent Gene Profiles of Sorbitol Non-Fermenting Shiga Toxin-Producing *Escherichia coli* Isolated from Goats in Southern Thailand

**DOI:** 10.3390/tropicalmed7110357

**Published:** 2022-11-07

**Authors:** Ratchakul Wiriyaprom, Ruttayaporn Ngasaman, Domechai Kaewnoi, Sakaoporn Prachantasena

**Affiliations:** Faculty of Veterinary Science, Prince of Songkla University, Songkhla 90110, Thailand

**Keywords:** sorbitol non-fermenting Shiga toxin-producing *Escherichia coli*, goats, southern Thailand, virulent genes

## Abstract

Shiga toxin-producing *Escherichia coli* (STEC) is the pathogenic *E. coli* causing disease in humans via the consumption or handling of animal food products. The high prevalence of these organisms in ruminants has been widely reported. Among STECs, O157 is one of the most lethal serotypes causing serious disease in humans. The present study investigated the prevalence of sorbitol non-fermenting STECs in goats reared in the lower region of southern Thailand and described the virulent factors carried by those isolates. Sorbitol non-fermenting (SNF)-STECs were found in 57 out of 646 goats (8.82%; 95% CI 6.75% to 11.28%). Molecular identification revealed that 0.77% of SNF-STEC isolates were the O157 serotype. Shiga toxin genes (*stx_1_* and *stx_2_*) and other virulent genes (i.e., *eae*A, *ehx*A, and *saa*) were detected by molecular techniques. The presence of *stx_1_* (75.44%) was significantly higher than that of *stx_2_* (22.81%), whereas 1.75% of the total isolates carried both *stx_1_* and *stx_2_*. Most of the isolates carried *ehx*A for 75.44%, followed by *saa* (42.11%) and *eae*A (12.28%). In addition, 21.05% of STEC isolates did not carry any *eae*A, *ehx*A, or *saa*. The first investigation on SNF-STECs in goat was conducted in the lower region of southern Thailand. The present study revealed that goats could be one of the potential carriers of SNF-STECs in the observing area.

## 1. Introduction

Bacterial foodborne infections are recognized as a public health concern, and the sources of bacterial contamination are generally related to animal feces [1]. *Escherichia coli* are Gram-negative bacteria with a high abundance in the environment, animal intestinal tract, and food [2]. Although most *E. coli* strains are not harmful to humans [2], certain pathogenic strains of *E. coli* can cause foodborne diseases. Shiga toxin-producing *E. coli* (STEC) are important foodborne agents that can cause serious symptoms [3], and previous outbreaks of STEC infection have been reported as sporadic and epidemic diseases [4]. The severity of STEC infection ranges from watery diarrhea to severe illness, such as hemorrhagic colitis and hemolytic–uremic syndrome [5]. The STECs were classified based on *E. coli* O antigen, with O157 and non-O157. Strain O157 is the causative agent of severe illness, with low infective doses in humans. Until now, more than 400 non-O157 STECs have been identified, and some of them are frequently related to human illness, such as O26, O45, O103, O111, O121, and O145 [6].

The use of sorbitol as a carbohydrate source is widely recommended for the screening of O157 STEC. Normally, most O157 STECs cannot use sorbitol and can be distinguished from the non-O157 strain [7]. However, sorbitol-negative phenotypes have been reported in certain non-O157 strains [8,9], and therefore, serological or molecular identification for O157 STEC should be performed in every sorbitol-negative *E. coli* isolate [10]. The PCR primers for *E. coli* O157 identification have been designed from the genetic element that encodes enzymes for O157 lipopolysaccharide biosynthesis [10]. Shiga toxin-producing *E. coli* has been identified by detecting genetic markers that encode Shiga toxins, i.e., *stx_1_* and *stx_2_* genes [11]. In addition, the other pathogenicity of STEC depends on the expression of genetic materials such as *eae*A, *saa*, and *ehx*A genes. During host cell attachment, the protein encoded by *eae*A gene generates the bond between the bacteria and the host cell, causing the structural change of the host cell cytoskeleton [12]. The *ehx*A is the plasmid-carried gene encoding enterohemolysin, which induces red blood cell destruction [13]. In addition, *saa* gene or STEC autoagglutinating adhesin is another virulent factor associated with severe disease [14,15]. 

Most STEC outbreaks are associated with the consumption of food of animal origin, particularly from cattle and other ruminants [11], and STEC-infected animals are responsible for carcass contamination in slaughterhouses [2]. According to previous studies, the occurrence of STEC in small ruminants is lower than that in cattle. In a study in Spain, 63.5% of a beef cattle herd were colonized by STEC, whereas 56.5% of a sheep herd were positive for STEC [16]. Similarly, a high prevalence of STEC has been reported for cattle in Brazil [17]. In Iran, STECs were isolated from 17.2% and 7.0% of goat and their carcasses, respectively [4]. 

Goats are widely raised in the south of Thailand, particularly in the southernmost part of the country [18]. However, only a few studies have described the prevalence of pathogenic *E. coli* in goats produced in Thailand. Thus, the present study investigates the prevalence of sorbitol non-fermenting STECs in goat herds in the south of Thailand and describes the virulent factors carried by those isolates.

## 2. Materials and Methods

### 2.1. Farm Description and Sample Collection 

**Figure 1:**
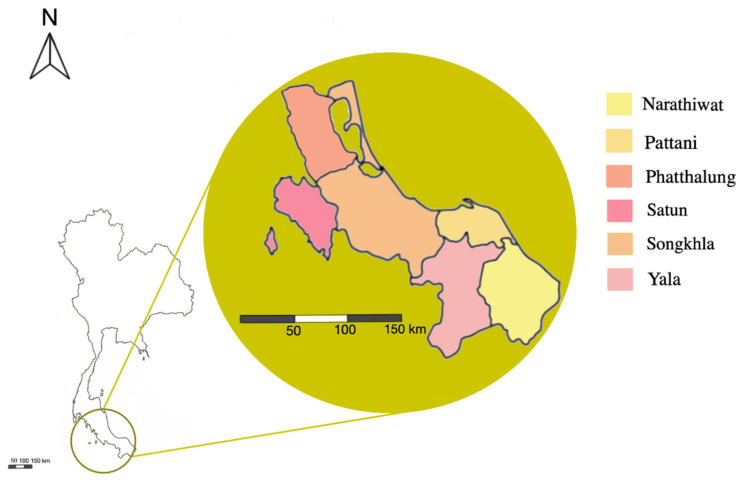
Location of goat farms participating in the present study. A total of 646 goats were selected from 61 goat farms in Narathiwat (*n* = 10), Pattani (*n* = 7), Phatthalung (*n* = 6), Satun (*n* = 10), Songkhla (*n* = 18), and Yala (*n* = 10).

From July 2020 to July 2021, the cross-sectional study was conducted in 61 goat farms located in six provinces of the lower region of southern Thailand i.e., Narathiwat, Pattani, Phatthalung, Satun, Songkhla, and Yala (Figure 1). Participating farms were selected from certified goat farms located in the target area. Most participating farms were small-holdings and raised the animals for meat production. The number of goats in investigated farms ranged between 5 to 1500. The sample size of the goats was calculated using a 50% expected prevalence, a 95% confident level, and a 5% margin of error. Thus, a total of 646 rectal swabs were randomly taken from healthy adult goats. Goats were selected based on inclusion criteria, i.e., over four months of age, good health condition and on exclusion criteria, i.e., late pregnancy, sick or injured animal (e.g., diarrhea, lethal wound, or zoonotic infection). Rectal swabs were taken from the animals by licensed veterinarians and well-trained staff. Permission for farm visits and data collection was obtained from all farm owners participating in this study. The samples were collected by inserting a sterile cotton swab into the animal’s anus. The tip of the cotton swab was gently rotated in the rectum to ensure the best representation in the sample. All samples were kept in Cary-Blair transport medium (M202; HiMedia, India) and sent to the laboratory for analysis within 4 h. 

### 2.2. Bacteriological Isolation and Primary Identification of Sorbitol Non-Fermenting Shiga Toxin-Producing Escherichia coli (SNF-STEC)

To screen SNF-STECs In samples, the swab tips were streaked on sorbitol–MacConkey agar (Difco^TM^, Becton, Dickinson and Company, Franklin Lakes, NJ, USA) and incubated at 37 °C for 18–24 h. Sorbitol non-fermenting colonies identical to *E. coli* (round, smooth, colorless appearance) were randomly selected for three to five colonies per sample. These colonies were sub-cultured on eosin–methylene blue agar (Merck KGaA, Darmstadt, Germany) at 37 °C for 18–24 h. Colonies with a greenish metallic sheen appearance were chosen for biochemical tests. Indole, methyl red, Voges–Proskauer, and citrate utilization tests (IMViC test) were conducted for all suspected isolates. The species *E. coli* was identified by indole-positive, methyl red-positive, Voges–Proskauer-negative, and citrate-negative results. Colonies biochemically confirmed as *E. coli* were stored in tryptic soy broth with 20% glycerol at −80 °C for further investigation as the sorbitol non-fermenting *E. coli*.

### 2.3. DNA Extraction

Presumptive bacterial isolates were suspended in distilled water and heated at 100 °C for 10 min. The bacterial mixture was then centrifuged at 13,000 rpm for 5 min to separate cell debris, and the supernatant was used as the DNA template for molecular techniques.

### 2.4. Serotyping and Virulent Gene Profiling by Molecular Techniques

Shiga toxin-producing *Escherichia coli* (STEC) was identified by the presence of Shiga toxin-encoding genes, i.e., *stx_1_* or *stx_2_* genes. The identification of the O157 stain was defined as the presence of the *rfb* (O antigen encoding) gene specific for *E. coli* O157 [19]. In addition, the virulent gene profile (i.e., *eae*A, *ehx*A, and *saa*) was characterized using primer pairs according to a previous study [20]. All primers used in the current study are shown in Table 1. Herein, 25 μL of the multiplex-polymerase chain reaction mixture (multiplex-PCR) contained 0.5 U of *Taq* DNA polymerase (KK5608, KAPA Biosystems, Darmstadt, Germany), 0.2 mM of deoxynucleoside triphosphates, 1.5 mM of MgCl_2_, and 0.5 μM of forward and reverse primers. The PCR conditions were as follows: 35 cycles of denaturation at 95 °C for 30 s, annealing at 54 °C for 50 s, and extension at 72 °C for 1 min. Agarose gel electrophoresis was conducted to determine the presence of the PCR product. MaestroSafe^TM^ (Labgene Scientific, Châtel-Saint-Denis, Switzerland) was used for DNA visualization in agarose gel under ultraviolet light.

### 2.5. Data Analysis

Sample size calculation and data analysis were conducted using the R Studio software (RStudio©, PBC, Boston, MA, USA). Differences between groups of interest were determined by the chi-squared test and Fisher’s exact test. Statistical significance was defined at *p* < 0.05.

## 3. Results

### 3.1. Prevalence of Sorbitol Non-Fermenting STECs (SNF-STECs) and O157 Serotype in Goat Herds

In this study, SNF-STECs were isolated from 57 out of 646 rectal swab samples from goats (8.82%; 95% CI 6.75% to 11.28%). The prevalence of SNF-STECs in Phatthalung was significantly higher than those in Songkhla, Narathiwat, Yala, and Pattani (*p* = 0.0033, *p* = 0.0048, *p* = 0.0003, and *p* < 0.0001, respectively). The proportion of SNF-STECs in Satun (15.63%) was also significantly higher than those found for Yala and Pattani (*p* = 0.0268 and *p* = 0.0098, respectively). In contrast, no statistical difference between the prevalence in Phatthalung and Satun was found. Among 61 participating goat farms, 24 found at least one animal testing positive for SNF-STECs (39.34%; 95% CI 27.07% to 52.69%). The molecular characterization of the O157 serotype revealed that goats were infected with O157 at a proportion of 0.77% (95% CI 0.10 to 1.45%). Of the O157-positive isolates, four were obtained from Songkhla and one from Narathiwat (Table 2). Thus, the prevalence of non-O157 SNF-STECs was 8.05% (95% CI 5.95% to 10.15%).

### 3.2. Virulent Gene Profiles of SNF-STEC Isolates

Among the Shiga toxin genes, *stx_1_* was most frequently detected (75.44%), followed by *stx_2_* (22.81%). Only one isolate from Songkhla (1.75%) carried both *stx_1_* and *stx_2_*. The proportion of stx_1_ in goats was significantly higher than that of *stx_2_* (*p* < 0.0001). The molecular detection of three virulent genes (i.e., *eae*A, *ehx*A, and *saa*) was conducted in 57 SNF-STEC isolates obtained from the goat samples. In general, the majority of the isolates carried *ehx*A (75.44%), followed by *saa* (42.11%) and *eae*A (12.28%) (Table S1). The presence of *ehx*A was significantly higher than that of *eae*A and *saa* (*p* < 0.0001 and *p* = 0.0006, respectively). A total of five virulent gene profiles were observed. Most isolates carried multiple virulent genes (49.12%), whereas single-gene carriers were detected in 29.82% of the isolates. Interestingly, 21.05% of the STEC isolates did not carry any of the observed genes. The virulent gene profile was dominated by *ehx*A–*saa* (36.84%), followed by *ehxA* (26.32%), *eae*A–*ehx*A (10.53%), and *saa* (3.51%). Only 1.75% of the isolates showed positive for three virulent genes, i.e., *eae*A, *ehx*A, and *saa* genes. A similar virulent gene profile was identified among O157 isolates (Table 3). 

## 4. Discussion

Shiga toxin-producing *Escherichia coli* strains (STECs) are widely recognized as a common cause of gastroenteritis in humans worldwide [2]. The STEC serotypes frequently involved in foodborne outbreaks are O157 STEC and non-O157 STEC, such as O26, O45, O103, O111, O113, O121, and O145 [4]. The O157 STEC is the main serotype related to severe illness in humans. Certain serotypes of non-O157 STEC have been reported in previous foodborne outbreaks [2]. Generally, the presence of sorbitol non-fermenting STECs is used to identify the O157 serotype [21], although certain strains of non-O157 STEC with a sorbitol-negative phenotype have been reported [22]. In the current study, the presence of non-O157 sorbitol non-fermenting STECs (SNF-STECs) indicates that the sorbitol-negative trait is not always the exclusive characteristic of the O157 serotype. To characterize the O157 serotype, phenotypic or genotypic confirmation is necessary [23].

In the current study, the prevalence of SNF-STECs and the O157 serotype in goats was 8.82% and 0.77%, respectively. In another study, Black Bengal goats in Bangladesh were infected with SNF-STECs in the proportion of 2.33% [24]. Furthermore, approximately 2.00% of goat fecal samples in the United Arab Emirates were positive for O157 STEC [25]. In South Africa, the O157 serotype was isolated from 3.46% of goat fecal samples [26]. Apart from studies in goats, SNF-STEC and O157 prevalence has been revealed in other ruminant species. In southern Thailand, the O157 serotype has been reported in bovine feces with a prevalence of 1.82% [27]. In the U.S., the occurrence of SNF-STECs in heifers during different seasons has been reported to range from 8.7% to 22.7% [28]. In another study, approximately 19.2% of cattle stools collected from a slaughterhouse in India were positive for non-sorbitol-fermenting *E. coli* [29]. In addition, healthy sheep in Turkey were infected with O157 *E. coli* with a prevalence of 9.1% [30]. The presence of foodborne pathogens in farm animals is probably associated with a risk of contamination in their successive meat products [31]. In the south of Thailand, contamination of beef with O157 STEC has been reported [32,33]. However, there is limited information regarding STECs or O157 *E. coli* in goat meat.

The prevalence of the *stx_1_* and *stx_2_* genes among the STEC-positive samples in the current study were 75.44% and 22.81%, respectively. Das Gupta and colleagues (2016) reported that the proportions of *stx_1_* and *stx_2_* genes detected in the STECs of goats were similar, whereas a study in South Africa found that the *stx_2_* gene was slightly more frequent than the *stx_1_* gene in goat fecal samples [26]. In contrast, several studies have reported that *stx_1_* is the predominant STEC gene in goat [34,35,36]. Regarding the STECs in cattle, previous studies have revealed a high prevalence of *stx_2_* genes compared to *stx_1_* genes [37,38]. Compared to the isolates that carried *stx_1_* genes or both *stx_1_* and *stx_2_* genes, *stx_2_*-positive isolates were more frequently associated with severe diseases in humans, such as hemorrhagic colitis and hemolytic–uremic syndrome [39]. A low prevalence of *eae*A has been reported in goat STECs [26,40]. In India, 16% of goat fecal samples carried the *eae*A gene [41]. Similarly, the *eae*A gene was detected in 12.28% of SNF-STEC isolates in this study. According to a previous study, *eae*A is involved in the production of adhesin, allowing bacteria to attach to the target cell [2]. Thus, the presence of *eae*A generally indicates a high virulence property in STEC isolates [12]. However, STECs without the *eae*A gene could be associated with severe human cases [14]. The STEC autoagglutinating adhesin (*saa*) gene have been detected in *eae*A-negative STECs that caused the outbreak of hemolytic–uremic syndrome or HUS [42]. In addition, hemolysin-encoding genes (i.e., *hly*A, *ehx*A, *she*A, and *e-hly*A genes) are related to the destruction of red blood cells during infection [43]. The current study revealed the prevalence of *ehx*A and *saa* genes at proportions of 75.44% and 42.11%, respectively. In another study, more than 50% of caprine STEC isolates in Vietnam carried *ehx*A and *saa* genes [35]. 

Because of the ethnic-based violence sporadically occurring in southernmost provinces of Thailand (i.e., Narathiwat, Pattani, and Yala), the limitation of the current study includes the difficulty of goat farm visitation and sample collection for these areas. Therefore, the rectal swab procedure had to be handled by staffs and veterinarians in the local area, not by researchers. However, the sample collection technique had been briefly explained to all staff prior to the beginning of the study. Due to security concerns, the sample size from each province had to be suitable for a one-day trip. Therefore, the proportion of the sample size might not conform to the actual goat population in these provinces. The reporting on provincial prevalence should be used with caution.

## 5. Conclusions

According to the low prevalence of SNF-STECs and the O157 serotype reported in the current study, goats might not be super-spreaders of these pathogens. However, further investigations are needed to determine the association between STECs isolated from goats and those from human cases. The potential virulent factors found in this study indicated that severe illness can be a result of the infection. To prevent the contamination of pathogenic *E. coli* in the food chain and the environment, abattoir employees should receive training on the fundamental ideas and standards of food and personal hygiene, as well as those specific to their particular food-processing operation, such as waste disposal, given that the spread of this pathogen causes human risk. Additionally, in order to avoid the release of waste into nearby streams, municipal authorities should relocate slaughterhouses away from streams and outfit the abattoirs with washing and garbage disposal facilities or similar.

## Figures and Tables

**Table 1:** Primer pairs used for the PCR reaction.

Primers	Target	Amplicon Size (Base Pair)	Reference
5′-CGGACATCCATGTGATATGG-3′	*rfb* gene for O157	259	[19]
5′-TTGCCTATGTACAGCTAATCC-3′			
5′-ATAAATCGCCATTCGTTGACTAC-3′	*stx_1_*	180	[19]
5′-AGAACGCCCACTGAGATCATC-3′			
5′-GGCACTGTCTGAAACTGCTCC-3′	*stx_2_*	255	[19]
5′-TCGCCAGTTATCTGACATTCTG-3′			
5′-GACCCGGCACAAGCATAAGC-3′	*eae*A	384	[20]
5′-CCACCTGCAGCAACAAGAGG-3′			
5′-GCATCATCAAGCGTACGTTCC-3′	*ehx*A	534	[20]
5′-AATGAGCCAAGCTGGTTAAGCT-3′			
5′-CGTGATGAACAGGCTATTGC-3′	*saa*	119	[20]
5′-ATGGACATGCCTGTGGCAAC-3′			

**Table 2:** Prevalence of SNF-STECs and the O157 serotype in goats according to province.

Provinces	Prevalence of (%)	
	SNF-STECs	O157 Serotype
Narathiwat	7.53	1.00
Pattani	2.13	Not found
Phatthalung	31.25	Not found
Satun	15.63	Not found
Songkhla	9.79	1.88
Yala	4.17	Not found

**Table 3:** Virulent genes profile of O157 isolates in goats.

Isolate’s Name	Farm Location	Virulent Genes				
		*stx_1_*	*stx_2_*	*eae*A	*ehx*A	*saa*
A1.9	Songkhla	Negative	Positive	Positive	Positive	Negative
E5.3	Songkhla	Negative	Positive	Positive	Positive	Negative
E5.4	Songkhla	Negative	Positive	Positive	Positive	Negative
E5.8	Songkhla	Negative	Positive	Positive	Positive	Negative
J5.9	Narathiwat	Negative	Positive	Positive	Positive	Negative

## Data Availability

Not applicable.

## Supplementary Materials

The following supporting information can be downloaded at: https://www.mdpi.com/article/10.3390/tropicalmed7110357/s1, Table S1: Summary data of Shiga toxin and virulent genes detected in isolates of the study.
